# Acute changes in glucose induced by continuous or intermittent exercise in children and adolescents with type 1 diabetes

**DOI:** 10.20945/2359-3997000000444

**Published:** 2022-03-23

**Authors:** Luis Paulo Gomes Mascarenhas, Valderi Abreu de Lima, Denise Barth Rebesco, Suzana Nesi França, Gabriel Ribeiro Cordeiro, Jorge Mota, Neiva Leite

**Affiliations:** 1 Universidade Estadual do Centro Oeste Departamento de Educação Física Irati PR Brasil Programa de Pós-graduação Interdisciplinar em Desenvolvimento Comunitário, Departamento de Educação Física, Universidade Estadual do Centro Oeste (UNICENTRO), Irati, PR, Brasil; 2 Universidade Federal do Paraná Departamento de Educação Física Curitiba PR Brasil Departamento de Educação Física, Núcleo de Qualidade de Vida, Universidade Federal do Paraná (UFPR), Curitiba, PR, Brasil; 3 Universidade Federal do Paraná Departamento de Pediatria Unidade de Endocrinologia Pediátrica Curitiba PR Brasil Unidade de Endocrinologia Pediátrica, Departamento de Pediatria, Universidade Federal do Paraná (UFPR), Curitiba, PR, Brasil; 4 Universidade do Porto Faculdade de Desporto Centro de Investigação em Actividade Física, Saúde e Lazer Porto Portugal Centro de Investigação em Actividade Física, Saúde e Lazer (CIAFEL), Faculdade de Desporto, Universidade do Porto, Porto, Portugal

**Keywords:** Type 1 diabetes, hypoglycemia, moderate-continuous exercise, high-intensity exercise

## Abstract

**Objective:**

To estimate the rate of change during exercise and during recovery in moderate-continuous exercise (MCE) and high-intensity intermittent exercise (HIIE) in children and adolescents with type 1 diabetes (T1D).

**Subjects and methods:**

Participants performed 2 sessions of exercise: thirty minutes of continuous activity on a cycle ergometer (60% of VO_2max_) and thirty minutes (60% VO_2max_) interspersed with five bouts of maximum intensity lasting ten seconds every five minutes. Capillary blood glucose was measured before and after each test. The glucose rate of change in exercise (RoC_E_) was calculated (final blood glucose – onset blood glucose/exercise time), and the glucose rate of change in recovery (RoC_R_) (blood glucose 30 minutes after exercise – end of exercise blood glucose/recovery time).

**Results:**

The study included thirty-one participants (aged 13 ± 1.88 years). A lower blood glucose reduction was observed in the HIIE group, as well as better recovery values before, after, and thirty minutes after the test, respectively (333.14 ± 69.53, 226.19 ± 68.05 and 201.77 ± 66.84 *versus* 211.36 ± 91.03, 155.98 ± 82,68 and 165.76 ± 72.94). Covariance analyses showed a significant difference in glycemic variation between continuous and intermittent protocols immediately after exercise (−2.90 *versus* −2.08) and during the recovery period (−0.677 *versus* −0.389).

**Conclusions:**

HIIE led to a lower glucose reduction rate per minute during exercise and better recovery in the first 30 minutes after exercise compared to MCE in children and adolescents with T1D.

## INTRODUCTION

In recent decades, several studies have shown that exercise in convergence with good glycemic control is an important agent in the prevention and reduction of pathologies associated with type 1 diabetes (T1D) ( 1 - 3 ). However, fear of hypoglycemia during and after exercise is still the main barrier to regular adherence to exercise practice by children and adolescents with T1D ( 4 , 5 ). In addition, the various forms of exercise can promote different responses in the glycemic level of these patients, since the response varies according to the type, duration, and intensity of activity ( 6 ). Another option available to people with T1D is the use of automated insulin delivery systems that can control glucose levels during and after exercise. Most of these are closed-loop hybrid systems, where the user manually adjusts target glucose and/or insulin infusion rates before starting exercise ( 7 ). However, these devices generally have a very high cost, making it impossible to generalize this equipment to all people with diabetes.

Continuous and moderate exercise apparently lead to reduced blood glucose in patients with T1D, through increasing the energetic demand for glucose by the skeletal muscles ( 5 ). Likewise, intermittent exercise, which reproduces the characteristics of the majority of children's daily activities, as well as some sports, seems to promote the release of counter-regulating hormones in order to stabilize the glycemic concentration during and after performance, collaborating to reduce the occurrence of acute hypoglycemia ( 8 ).

Despite advances in this area of knowledge, uncertainties remain regarding the acute impact of different types of exercise on the glycemic control of children and adolescents with T1D, especially on deficient glycemic control. García-García and collaborators (2015) suggested a rate to characterize the mean trend of change in glucose (RoC) in order to estimate the acute changes induced by exercise ( 9 ).

Thus, the current study aimed to estimate the rate of change acutely induced during exercise (RoC_E_), and rate of change in the initial recovery period (RoC_R_), after moderate-continuous exercise (MCE) and high-intensity intermittent exercise (HIIE) in children and adolescents with T1D.

## SUBJECTS AND METHODS

This cross-sectional descriptive study evaluated 31 patients with T1D, treated at the Pediatric Endocrinology Unit of the school hospital of the Federal University of Paraná. The sample was selected for convenience, with evaluation of the patients who agreed to participate in the research and who received authorization from the parents or guardians, upon presentation of the Free and Informed Consent Term, in accordance with Resolution 196/96 of the National Health Council. Approved by the Plataforma Brasil, CAAE number 44193214-7.0000.0096.

Children and adolescents diagnosed with diabetes for at least two years, aged 10 to 15 years, were included. Patients with orthopedic alterations or other comorbidities that made physical activity impossible were not included.

The techniques described by Lohman (1992) were used to obtain anthropometric measurements ( 10 ). Stature was measured with a portable vertical stadiometer (WCS^®^, Brazil), staggered in 0.1 cm units and evaluated in centimeters (cm), at the end of maximum inspiration. Body mass was measured on a portable digital scale (Filizola^®^, Brazil), in kilograms (kg). The body mass index (BMI) was calculated using the formula: body weight (kg) ÷ height (m) X height (m). To calculate the z score of the BMI, the WHO Antro Plus 1.0.4 program was used.

The Bouchard and cols. (1983) questionnaire was used to assess the level of physical activity (PAL-MVPA / DAY), which consists of recording the individual's daily activities every 15 minutes, for three days (two days a week and one weekend). The instrument enables estimation of the energy expenditure by the average of the days recorded ( 11 ).

Glycemic control was evaluated using glycated hemoglobin (HbA1c) levels. Blood was collected through venous puncture, after previous fasting of 12 hours, and then taken for analysis.

The evaluated patients visited the laboratory four times. The first visit aimed to obtain anthropometric measurements and blood collection. On the second visit, participants performed the cardiorespiratory fitness test (VO_2max_), using the K4b2^®^ portable gas analyzer (Cosmed^®^, Italy). The Balke protocol adapted to a cycle ergometer was used ( 12 ), starting with a load of 25 Watts and a speed of 50 RPM and increasing by 25 watts every three minutes until the maximum heart rate was reached ( 13 ), or the participant was unable to maintain the speed and load.

After 48 hours, the participant returned to the laboratory to perform the MCE test. The protocol consisted of 30 minutes of continuous activity on a cycle ergometer with an intensity of 60% of the maximum VO_2max_.

After an interval of one week, participants returned to the laboratory to perform the HIIE test. The participant pedaled for 30 minutes on a cycle ergometer with a load of 60% of the maximum VO_2max,_ intercalated with five sprints of maximum intensity lasting ten seconds every five minutes. Capillary blood glucose was measured before, immediately after, and 30 minutes after the end of the test. The insulin therapy of the patients was not altered, with mean daily doses of 26.5 ± 7.36 long-acting insulin (Glargine) and doses of 7.5 ± 3.41 ultrarapid-acting insulin (Aspart and Lispro). All participants used both types of insulin, long-acting and fast-acting, and there were no changes in pre-exercise doses in either protocol.

Pre-exercise meals were individually prepared by nutritionists and provided in the laboratory, including between 30% and 35% of the daily caloric requirement, of which 50 to 55% was composed of carbohydrates, according to Murphy and Barr (2006) ( 14 ). The pre-exercise meal was always provided 30 minutes before the beginning of activities.

The magnitude of variation in glucose per minute during exercise (RoCE) was calculated to characterize the mean trend of glucose rate of change (RoC) during the performance of exercise. The calculation included the average glucose between the start and end of the exercise session, and the duration of the exercise. Additionally, in order to assess the mean RoC in the recovery phase (i.e. immediately after exercise termination), we calculated the RoC_R_ index over a recovery interval of 30 min post-exercise.

The glucose rate of change in exercise (RoC_E_) was calculated as follows (final blood glucose – onset blood glucose/exercise time), and the glucose rate of change in recovery (RoC_R_) as follows (blood glucose 30 minutes after exercise – end of exercise blood glucose/recovery time).

Statistical analysis was performed using the SPSS program for Windows, version 22. Descriptive statistics with mean and standard deviation were used for the main sample characteristics. The Student T-test was used for sex comparisons. For the glycemic change data, the Shapiro-Wilk normality test was performed. The inter-group glycemic change was compared using Analysis of Covariance (ANCOVA), because it can be compared considering the variability of other variables. The analyses were carried out with and without insulin dose adjustment, adopting a significance level of p < 0.05. The sampling power was calculated posteriorly in G * Power software (see 3.1.9.2), using the ANCOVA test. An effect size of 0.25, α of 0.05, and total sample of 31 participants were assigned, giving a statistical power of 0.91.

## RESULTS

The descriptive data are shown in Table 1 . The final sample consisted of 31 adolescents (15 boys and 16 girls). There were no statistically significant differences between sexes in the variables of interest.

**Table 1:** Sample characterization

	Total (N = 31)	Male (N = 15)	Female (N = 16)	p
Age (years)	13.08 ± 1.88	13.43 ± 1.93	12.78 ± 1.85	0.354
T1D diagnosis (years)	6.43 ± 3.80	6.02 ± 3.59	6.85 ± 4.11	0.605
Weight (kg)	48.66 ± 13.30	47.55 ± 13.15	49.77 ± 13.85	0.667
Height (cm)	124.16 ± 66.08	125.92 ± 68.51	122.40 ± 66.10	0.891
BMI	19,65 ± 2,79	19,36 ± 2,62	19,87 ± 2,98	0,627
BMI z score	0,13 ± 0,93	-0,15 ± 0,92	0,38 ± 0,89	0,142
HbA1c (%)	9.74 ± 1.59	9.82 ± 1.83	9.65 ± 1.38	0.783
PAL-MVPA /DAY	27,35 ± 6,95	34,88 ± 6,11	36,30 ± 2,50	0,404

HbA1c: glycated hemoglobin. PAL-MVPA /DAY: time in minutes of moderate to vigorous physical activity per day.

Table 2 shows absolute mean values of glycemic change before, immediately after, and thirty minutes after the end of the exercise tests. According to the mean blood glucose values (before, immediately after, and thirty minutes after the tests), there was a lower reduction in blood glucose in the mean values for the intermittent protocol and a tendency for glucose to rise during the recovery period, while in the continuous protocol there was a decrease in blood glucose.

**Table 2:** Mean blood glucose values (before, immediately after, and thirty minutes after the exercise tests)

a	Before (mg/dL)	After (mg/dL)	Δ % *
MCE	333.14 ± 69.53	226.19 ± 68.05	-32.10
HIIE	211.36 ± 91.03	155.98 ± 82.68	-26.20
**b**	**After (mg/dL)**	**30 min after (mg/dL)**	
MCE	226.19 ± 68,05	201.77 ± 66.84	-10.80
HIIE	155.98 ± 82.68	165.76 ± 72.94	6.27
**c**	**Before (mg/dL)**	**30 min after (mg/dL)**	
MCE	333.14 ± 69.53	201.77 ± 66.84	- 39.4
HIIE	211.36 ± 91.03	165.76 ± 72.94	-21.5

* Delta variation (%) of mean blood glucose values. a: Comparison of glycemia assessed before and at the end of exercise. b: Comparison of glycemia assessed at the end and 30 minutes after exercise. c: Comparison of glycemia assessed before and 30 minutes after exercise.

The ANCOVA analysis showed a difference between the Continuous and Intermittent exercise protocols regarding the RoC_E_, with a greater decrease in glucose levels found in the continuous protocol even after adjusting for insulin applied before the exercise ( Table 3 ). Furthermore, our data also showed that a lower RoC_R_ for intermittent exercise, demonstrating better recovery of glycemic levels after this type of exercise even after adjusting for insulin doses ( Table 4 ).

**Table 3:** RoC_E_ Comparison in the Continuous and Intermittent exercise protocols with and without adjustment by insulin dose

Test	n	Mean (mg/dL/min)	CI95%	p
MCE	31	-2.90	(−3.41; −2.39)	0.026 *
HIIE	31	-2.08	(−2.59; −1.57)	
MCE a	31	-2.89 a	(−3.42; −2.37)	0.036 *
HIIE a	31	-2.09 a	(−2.61; −1.57)	

* p-value for ANCOVA; CI 95%: 95% confidence interval;

a Adjusted mean by insulin dose applied. (RoC_E_) Glucose rate of change in exercise.

**Table 4:** RoC_R_ comparison in Continuous and Intermittent exercise protocols with and without adjustment by insulin dose

Test	n	Mean (mg/dL/min)	CI95%	p
MCE	31	-0.677	(−1.03; −0.32)	0.001 *
HIIE	31	-0.389	(−0.03; −0.74)	
MCE a	31	-0.715 a	(−1.07; −0.35)	0.001 *
HIIE a	31	-0.426 a	(−0.06; −0.78)

* p-value for ANCOVA; CI 95%: 95% confidence interval;

a Adjusted mean by insulin dose applied. (RoC_R_) Glucose rate of change in recovery.

## DISCUSSION

This study assessed the acute impact of two types of structured exercise (MCE and HIIE) on glycemic levels in children and adolescents with T1D. The rate of change was quantified both during the exercise and in the recovery period. Our results suggest that compared to continuous exercise, intermittent exercise can reduce the risk of hypoglycemia during practice and immediately after.

Indeed, our data showed that HIIT led to a lower RoC_E_ compared to continuous exercise, with a significant difference between protocols. The mean values of glucose in the initial blood collection were significantly higher in the continuous protocol; however, many other studies corroborate the current finding of a lower decrease in glycemia related to intermittent exercise when compared to continuous exercise protocols, with a statistically significant difference ( 15 , 16 ). Thus, the results need to be interpreted with caution, as this initial difference in glucose may explain the results found only in this research.

This lower change in blood glucose is in line with Moser and cols. (2015), who reported a lower glycemic reduction during intermittent exercise on a cycle ergometer, when evaluating seven trained male subjects with T1D. Thus, intermittent exercise seems to be a strategy when the objective is to reduce the risk of exercise-induced hypoglycemia ( 15 ).

Lactate and glycerol levels were not evaluated in the present study, however high levels may be related to better glucose stabilization and better post-exercise recovery induced by a higher intensity of intermittent exercise ( 16 ).

Elevated lactate after exercise has been proposed as a gluconeogenesis stimulus that can attenuate declines in blood glucose, or as a glucose competitor agent by reducing muscle glucose uptake during exercise ( 17 , 18 ).

A study evaluating the effects of exercise in T1D patients with glargine/glulisine use found that HIIE limited BG decreases during and following exercise and therefore offered strategic choices in the prevention of exercise-induced hypoglycemia. In this case, the participants performed 60 minutes of exercise at 50% VO_2peak_ in the postprandial state under three different conditions: no pre-exercise glucose intake; 30g pre-exercise glucose intake; and no pre-exercise glucose intake, but with the inclusion of high-intensity sprints. The results suggest that intermittent exercise may be an alternative to glucose use in terms of preventing hypoglycemia without excessive increases in pre-exercise glucose ( 16 ).

The fact that intermittent exercise causes lower changes in blood glucose and, consequently, less hypoglycemia, may be associated with higher endogenous glucose production. Guelfi and cols. (2007) evaluated endogenous glucose production in intermittent and continuous exercise protocols and found higher endogenous glucose production in the intermittent exercise group, which minimizes the risk of hypoglycemia ( 17 ).

The patients’ concern about the risk of hypoglycemia exists both during exercise and after practice. In this study, better recovery of glucose levels expressed by RoC_R_ was observed for intermittent exercise. The lower change in blood glucose 30 minutes after intermittent exercise may be associated with increased secretion of counter-regulating hormones such as cortisol, glucagon, and catecholamines, causing increased glucose production by the liver ( 18 ).

Future research should be conducted with control groups and including analysis of hormone responses during and after exercise practice, in order to better clarify the effects of intermittent exercise on glycemic changes in children and adolescents, as well as the benefits and possible risks. Further studies are important to provide more evidence-based data on this topic so that children and adolescents can safely take advantage of the practice of intermittent exercise, as this type of exercise presents characteristics similar to various sports and spontaneous activities normally practiced by children and adolescents.

In conclusion, intermittent exercise led to a lower glucose reduction rate per minute during exercise and better recovery in the first 30 minutes after exercise compared to continuous exercise in children and adolescents with T1D.
